# High-Fiber Diet Supplemented with N-Carbamylglutamate Modulates Uterine Microbiota, Metabolites, and Transcriptome to Improve Reproductive Efficiency in Sows

**DOI:** 10.3390/antiox15050542

**Published:** 2026-04-24

**Authors:** Yaxu Liang, Hongyang Wang, Zhibo Wang, Yingying Zhang, Weilong Tu, Jieke Zhou, Yuduan Diao, Huijie Pei, Ji Huang, Xiang Zhou, Yongsong Tan

**Affiliations:** 1 Institute of Animal Husbandry & Veterinary Science, Shanghai Academy of Agricultural Sciences, Shanghai 201106, China; 20250603@saas.sh.cn (Y.L.); wanghongyang@saas.sh.cn (H.W.); zhangyingying@saas.sh.cn (Y.Z.); tuweilong@saas.sh.cn (W.T.); zhoujieke@saas.sh.cn (J.Z.); 20220603@saas.sh.cn (Y.D.); huijiepei@saas.sh.cn (H.P.); android717@gmail.com (J.H.); 2 Shanghai Engineering Research Center of Breeding Pig, Shanghai 201106, China; 3 Shanghai Animal Disease Prevention and Control Center, Shanghai 201103, China; 2020205010@stu.njau.edu.cn; 4 College of Animal Science and Technology, Huazhong Agricultural University, Wuhan 430070, China; 5 Hubei Hongshan Laboratory, Wuhan 430070, China

**Keywords:** high-fiber diet, N-carbamylglutamate, multi-omics analysis, antioxidant, reproductive performance

## Abstract

Uterine microbiome homeostasis and antioxidant capacity are critical for sow fertility. While high-fiber diets and N-carbamylglutamate (NCG) individually enhance sow fertility, their synergistic effects on the antioxidant status, microbiota, metabolites, and transcriptome remain unclear. Here, sows were assigned to the low-fiber (3.73%) or high-fiber (7.46% crude fiber) group, each without or with 0.05% NCG, throughout the 114-day gestation. Sex hormones and antioxidants in serum were detected. Multi-omics approaches were employed to investigate the impact of a high-fiber diet supplemented with NCG (H + N) on uterine microbiota, metabolites, and gene expression profiles. The study revealed that H + N significantly increased total antioxidant capacity (T-AOC) level in serum. Metagenomic analysis revealed an increased abundance of *Clostridium disporicum* in the uterine microbiota. Plasma metabolomics identified hydroxylysine as a key metabolite mediating this effect, and this metabolite was positively correlated with elevated abundance of *Clostridium disporicum*. Subsequent transcriptomic profiling revealed activation of the PI3K-Akt signaling pathway, closely linked to improved T-AOC level. Overall, these findings demonstrated that H + N could modulate the uterine microbiota (specifically *Clostridium disporicum*), increase hydroxylysine production, and activate the PI3K-Akt signaling pathway. These effects further enhanced hormonal activity and antioxidant capacity, ultimately improving sow reproductive efficiency.

## 1. Introduction

Uterine microenvironment is pivotal for mammalian reproductive function. Disrupted uterine microbiome and excessive oxidative stress are detrimental to reproductive performance [1,2]. Therefore, nutritional interventions designed to optimize uterine microbiome and redox balance have become promising approaches to enhance reproductive efficiency in swine [3].

Dietary fiber (DF), a crucial component in swine diet, has been demonstrated to modulate microbiota composition and tryptophan metabolism and improve insulin sensitivity, thereby contributing to improved reproductive outcomes [4,5]. Supplemental DF during gestation could improve reproductive performance by improving oocyte quality, accelerating oocyte maturation, and promoting uterine development [3,6,7]. Concurrently, N-carbamylglutamate (NCG), a potent activator of endogenous arginine biosynthesis, has emerged as a promising nutritional strategy to improve reproductive performance in chickens (1%) and mice (0.1%) [8,9]. In sows, dietary supplementation with 0.05% NCG has been shown to enhance the development of reproductive tissues including the endometrium and placentae [10]. Furthermore, 0.05% NCG could significantly increase litter size and proportion of viable offspring in sows by regulating energy metabolism, lipid synthesis, and glutathione homeostasis, a major intracellular antioxidant system [10]. However, the potential synergistic effects between DF levels and NCG on uterine microbiota, antioxidant status, and reproductive function require further elucidation.

Mounting evidence has confirmed the presence of distinct microbial communities in the female reproductive tract, especially in the uterus [11,12]. These low-biomass but functionally important communities are critical for maintaining reproductive homeostasis in sows [13,14,15]. Recent studies have further suggested that reproductive tract microbiota modulates local redox balance, suggesting a potential microbiota–antioxidant–reproduction axis [16,17,18]. However, whether DF and NCG improve reproductive performance via uterine microbiota-dependent mechanisms remains to be explored.

In the present study, Landrace x Yorkshire sows were selected to investigate the synergistic effects of elevated DF (achieved through supplementation with soybean bran and beet pulp) and 0.05% NCG [10] on reproductive performance. The establishment and maintenance of pregnancy are tightly regulated by reproductive hormones. Progesterone is also essential for pregnancy progress [19]. Disruption of progesterone signaling results in pregnancy termination and the induction of parturition [19,20]. Therefore, serum sex hormone levels and systemic antioxidant capacity were assessed. To comprehensively elucidate the underlying mechanisms through which the high-fiber diet supplemented with 0.05% NCG (H + N) enhances sow reproduction, multi-omics approaches were employed, integrating metagenomic, metabolomic, and transcriptomic analyses.

## 2. Materials and Methods

### 2.1. Animals, Diets, and Experimental Design

All animal procedures were carried out in accordance with the guidelines approved by the Ethics Committee of the Shanghai Academy of Agricultural Sciences (approval no. SAASPZ0524108). From Hunan Longhua Agriculture and Animal Husbandry Technology Co., Ltd. (Zhuzhou, China), a total of 400 healthy Landrace × Yorkshire sows with identical parity (second or third) were chosen and assigned to receive either a low-fiber (LF) or high-fiber (HF) diet. The sows were randomly divided into four treatment groups (*n* = 100/group): LF diet without NCG (L − N; 3.73% crude fiber), LF diet supplemented with NCG (L + N; 3.73% crude fiber + 0.05% NCG), HF diet without NCG (H − N; 7.46% crude fiber), and H + N (7.46% crude fiber + 0.05% NCG). The L − N group was designated as the basal control, representing the DF level conventionally used in swine production [21].

To elevate fiber levels in diets, corn was partially replaced with soybean bran and beet pulp. Detailed information on the ingredient composition and nutrient profiles of the experimental diets is presented in Table 1 [22]. The experiment began on the breeding day and covered the entire 114-day gestation period. Feedings were administered twice a day, at 06:30 and 16:30 h. Feed intake was modulated based on pregnancy progression: 2.0 kg·day^−1^ in the initial phase (days 0–30), 2.5 kg·day^−1^ in the middle phase (days 31–90), and 3.5 kg·day^−1^ in the final phase (days 91–110). Intake was subsequently reduced, reaching 1.0 kg on the day of farrowing [23,24].

### 2.2. Sample Collection

After the 114-day feeding trial, blood samples (approximately 10 mL per sow) were collected from the tail vein of 10 randomly selected sows per group at approximately 08:00 h. Serum was isolated by centrifugation at 3000× *g* for 10 min. The resulting serum was preserved at −20 °C for further analysis. Following this, six sows per treatment group were randomly chosen for euthanasia, and endometrium and cervix samples were collected.

### 2.3. Detection of Serum Indicators

The concentrations of cortisol, estradiol, and progesterone in sows were quantified using commercial assay kits (Cayman Chemical, Ann Arbor, MI, USA; Cat. No. 500360, 501890, and 582601, respectively). The total antioxidant capacity (T-AOC) and superoxide dismutase (SOD) activity were determined with kits obtained from Nanjing Jiancheng Bioengineering Institute (Cat. No. A015-3-1 and A001-1). All assays were performed strictly in accordance with the manufacturers’ instructions.

### 2.4. Metagenomics Sequencing

Approximately 0.3 g of endometrium and cervix was accurately weighed, and total microbial genomic deoxyribonucleic acid (DNA) was extracted using the Mag Bind Soil DNA Kit (catalog no. M5635-02; Omega Bio-Tek, Norcross, GA, USA) following the manufacturer’s instructions. DNA concentration was assessed using a Qubit™ 4 Fluorometer (Invitrogen, Waltham, MA, USA), while integrity was evaluated via 1% agarose gel electrophoresis. Only samples meeting the quality thresholds (DNA concentration > 12.5 ng·μL^−1^, and a dominant electrophoretic band > 20 kb) were selected for subsequent library preparation. Genomic DNA was fragmented to 200–400 bp using an ultrasonicator. An end-repair reaction was performed to generate blunt-ended double-stranded DNA, followed by adenylation of the 3′ ends to facilitate adapter ligation. An adapter ligation reaction was then assembled to attach adapters to the DNA fragments. The ligated products were amplified using a prepared polymerase chain reaction (PCR) reaction system. Subsequently, the amplified products were denatured to single strands and circularized. Remaining linear DNA was digested enzymatically. Qualified metagenomic libraries were sequenced on the NovaSeq X Plus platform (Illumina, San Diego, CA, USA) in a paired-end 150 bp mode at Personalbio Co., Ltd. (Shanghai, China).

### 2.5. Metabolomics Sequencing

Metabolomics profiling was conducted by integrating chromatography and mass spectrometry (MS) technology. Metabolite extraction was performed as follows: approximately 50 mg of each sample was homogenized using a tissue homogenizer (55 Hz, 60 s, repeated twice). Then, 800 μL of methanol/acetonitrile solution (1:1, *v*/*v*) was added to precipitate proteins. The mixture was ultrasonicated for 30 min and subsequently incubated at −20 °C for 30 min. After centrifugation (12,000 rpm, 4 °C, 10 min), the supernatant was collected and vacuum-dried. The dried metabolite was reconstituted in 150 μL of 50% methanol containing 5 ppm 2-chlorophenylalanine. The solution was centrifuged again under the same conditions, and the resulting supernatant was filtered through a 0.22 μm membrane into a detection bottle.

The chromatographic separation was performed on an ACQUITY UPLC HSS T3 column (100 Å, 1.8 µm, 2.1 mm × 100 mm) with a flow rate of 0.4 mL·min^−1^. The column temperature was at 40 °C, and the auto-sampler was set at 8 °C.

Mass spectrometric analysis was performed on a Thermo Orbitrap Exploris 120 (Thermo Scientific, Bremen, Germany) instrument controlled by Xcalibur software (v4.7, Thermo Scientific). Data was acquired in data-dependent acquisition mode, switching between positive and negative ionization modes. A heated electrospray ionization source was employed with the following parameters: spray voltage of 3.5 kV (positive) or −3.0 kV (negative), sheath gas flow rate of 40 (arbitrary units), auxiliary gas flow rate of 15 (arbitrary units), capillary temperature of 325 °C, and auxiliary gas heater temperature of 300 °C. Full MS scans were acquired at a resolution of 60,000 over the m/z range of 100–1000, with an automatic gain control target set to “Standard” and a maximum injection time (Max IT) of 100 ms. The top 4 most intense ions from each full scan were selected for fragmentation. MS/MS spectra were generated at a resolution of 15,000 using higher-energy collisional dissociation with a normalized collision energy of 30%.

### 2.6. Transcriptome Sequencing

Total ribonucleic acid (RNA) was extracted from endometrium using the Trizol method. Total RNA (≥1 μg) was used for strand-specific library construction using the NEBNext Ultra II RNA Library Prep Kit for Illumina (New England Biolabs, Ipswich, MA, USA) following the manufacturer’s instructions. Poly(A) + messenger ribonucleic acid (mRNA) was enriched with Oligo(dT) magnetic beads and fragmented using divalent cations under elevated temperature. Fragmented mRNA was used as a template to synthesize first-strand complementary DNA (cDNA) with random primers, followed by second-strand synthesis to generate double-stranded cDNA. The cDNA was subjected to end repair, followed by 3′ adenylation and adapter ligation. Libraries with sizes of approximately 400–500 bp were selected via AMPure XP beads, subjected to PCR amplification, and purified again with AMPure XP beads to generate the final sequencing-ready library. Library quality was assessed on an Agilent 2100 Bioanalyzer with the High Sensitivity DNA Kit (Agilent Technologies, Santa Clara, CA, USA). Quantification was performed via PicoGreen fluorescence measurement using a Quant-iT PicoGreen dsDNA Assay Kit (Invitrogen, Carlsbad, CA, USA) on a Promega Quantifluor fluorometer (E6090). Effective library concentration was determined by quantitative real-time polymerase chain reaction (qPCR) using a StepOnePlus Real-Time PCR System (Thermo Fisher Scientific, Waltham, MA, USA). Multiplexed libraries were normalized, pooled in equimolar ratios, and sequenced on an Illumina platform in PE150 mode.

### 2.7. Statistical Analysis

In metagenomic analysis, a correlation network was constructed among KEGG Orthology (KO) entries that exhibited significant alterations (*p* < 0.05) between the L − N and H + N groups, using a Spearman correlation threshold of |r| > 0.9 to define high correlations. In metabolomics, random forest analysis was performed using the mlr3verse package (version 0.2.7). Subsequently, Receiver Operating Characteristic (ROC) curves were generated with the pROC package (version 1.18.2) to identify key metabolites within the differential set. KEGG enrichment analysis of differential metabolites was conducted using the clusterProfiler package (version 4.6.0). In transcriptomic studies, differential gene expression was assessed with DESeq2 (version 1.38.3). Genes with |log_2_(fold change)| > 1 and an adjusted *p*-value < 0.05 were defined as differentially expressed.

Statistical analysis was performed using IBM SPSS Statistics software (version 22.0). The normality of the data was assessed using the Kolmogorov–Smirnov test. Data that were not normally distributed underwent a log-transformation. Subsequently, a two-way analysis of variance was employed to evaluate the main effects of DF level and NCG supplementation, as well as their interaction effect. In the event of a significant effect, post hoc comparisons among treatment groups were conducted using Duncan’s multiple range test. All data were presented as mean ± standard error of the mean (SEM). Differences were considered statistically significant at *p* < 0.05. In figures, significance is indicated either with asterisks (* *p* < 0.05, ** *p* < 0.01, *** *p* < 0.001) or with different superscript letters.

## 3. Results

### 3.1. Effects of Dietary Fiber and N-Carbamylglutamate on Serum Sex Hormones and Antioxidant Level

**Figure 1:**
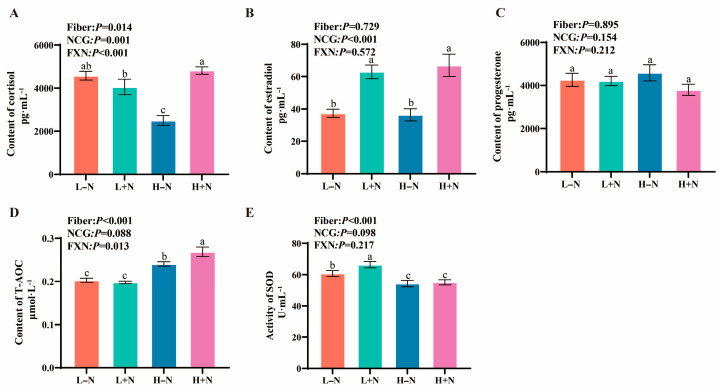
Effects of dietary fiber and N-carbamylglutamate on serum sex hormones and antioxidant level in pregnant sows. (**A**–**C**): Comparisons of serum sex hormones among treatment groups (L − N, L + N, H − N, and H + N): (**A**) content of cortisol (pg·mL^−1^), (**B**) content of estradiol (pg·mL^−1^), and (**C**) content of progesterone (pg·mL^−1^). (**D**,**E**): Comparisons of total antioxidant capacity (T-AOC; μmol·L^−1^) and activity of superoxide dismutase (SOD; U·mL^−1^) among treatment groups (L − N, L + N, H − N, and H + N). F: fiber; NCG: N-carbamylglutamate; L − N: low-fiber diet without NCG; L + N: low-fiber diet supplemented with NCG; H - N: high-fiber diet without NCG; H + N: high-fiber diet supplemented with NCG. FXN: interaction between fiber and NCG. ^a–c^ Values with different superscript letters (a–c) indicate significant differences among treatments (*p* < 0.05), while values sharing the same letter indicate no significant difference (*p* > 0.05).

Levels of sex hormones and antioxidants in sow serum samples were presented in Figure 1. Contents of cortisol (Figure 1A) and T-AOC (Figure 1D), as well as activity of and SOD (Figure 1E) were significant effects by fiber level in diet. NCG supplementation significantly influenced contents of cortisol and estradiol (Figure 1A,B). In detail, HF diet significantly increased T-AOC content (Figure 1D), accompanied by a decrease in cortisol content (Figure 1A). NCG supplementation resulted in a significant elevation of cortisol and estradiol concentrations (Figure 1A,B). Moreover, significant interactions between DF and NCG supplementation were detected, affecting contents of cortisol and T-AOC (Figure 1A,D).

### 3.2. Dietary Fiber and N-Carbamylglutamate Reshape Uterine Microbiota

**Figure 2:**
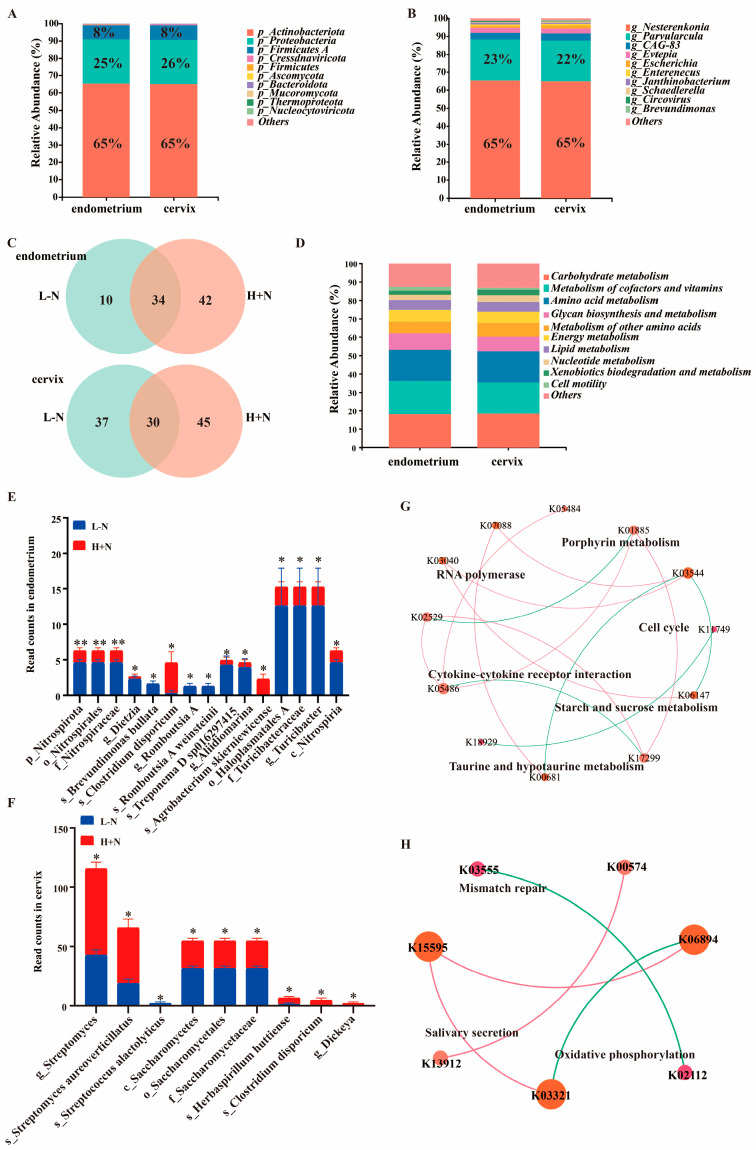
Effects of dietary fiber and N-carbamylglutamate on uterine microorganisms. (**A**,**B**): Bar charts depicted microbial composition at phylum (**A**) and genus (**B**) levels in endometrium and cervix. (**C**): Venn diagram illustrated number of unique and shared microorganisms at species level between L − N and H + N groups in endometrium and cervix. (**D**): Bar chart depicted microbial functions in endometrium and cervix. (**E**,**F**): Read counts of differentially abundant microorganisms between L − N and H + N groups in endometrium (**E**) and cervix (**F**). (**G**,**H**): Highly correlated network of significantly changed KEGG Orthology (KO) of metagenome between L − N and H + N groups in endometrium (**G**) and cervix (**H**). *p* < 0.05, Spearman’s |r| > 0.9. L − N: low-fiber diet without N-carbamylglutamate (NCG); L + N: low-fiber diet supplemented with NCG; H − N: high-fiber diet without NCG; H + N: high-fiber diet supplemented with NCG. Asterisks indicate the level of statistical significance: * *p* < 0.05 and ** *p* < 0.01.

To investigate effects of DF and NCG on uterine microbiota, metagenomic sequencing was conducted. At the phylum level, the microbiota in both the endometrium and cervix was predominantly composed of Actinobacteriota, Proteobacteria, and Firmicutes A (Figure 2A). At the genus level, Nesterenkonia and Parvularcula were dominant microbial taxa in the endometrium and cervix (Figure 2B). Venn diagram analyses demonstrated that compared to L − N, H + N significantly altered the microbial composition in both endometrium and cervix (Figure 2C). Functional analysis revealed that microbiota in endometrium and cervix was primarily associated with carbohydrate metabolism, metabolism of cofactors and vitamins, amino acid metabolism, glycan biosynthesis and metabolism, energy metabolism, lipid metabolism, and nucleotide metabolism (Figure 2D). DF levels and NCG both had significant effects on microbiota in the endometrium and cervix (Figure S1A–D). At species level, compared to L − N group, H + N treatment significantly increased abundance of *Clostridium disporicum* in endometrium (Figure 2E). Additionally, the H + N group exhibited significantly increased abundances of *Streptomyces aureoverticillatus*, *Herbaspirillum huttiense*, and *Clostridium disporicum* in the cervix compared to L − N group (Figure 2F).

Moreover, network analysis based on robust and significant correlations revealed that significantly changed KO in the endometrium were predominantly centered on pathways associated with metabolism and cell growth. These included starch and sucrose metabolism, taurine and hypotaurine metabolism, porphyrin metabolism, and the cell cycle (Figure 2G). In the cervix, the identified networks primarily included oxidative phosphorylation, mismatch repair, and salivary secretion (Figure 2H). These results demonstrated that the combination of high-fiber diet and NCG supplementation exerts beneficial effects on the uterine microbial ecosystem of the experimental units. Specifically, this treatment increased beneficial bacteria (e.g., *Clostridium disporicum*) [26] and enhanced metabolic pathways related to carbohydrate and energy metabolism.

### 3.3. Dietary Fiber and N-Carbamylglutamate Modulate Uterine Metabolite Profiles

**Figure 3:**
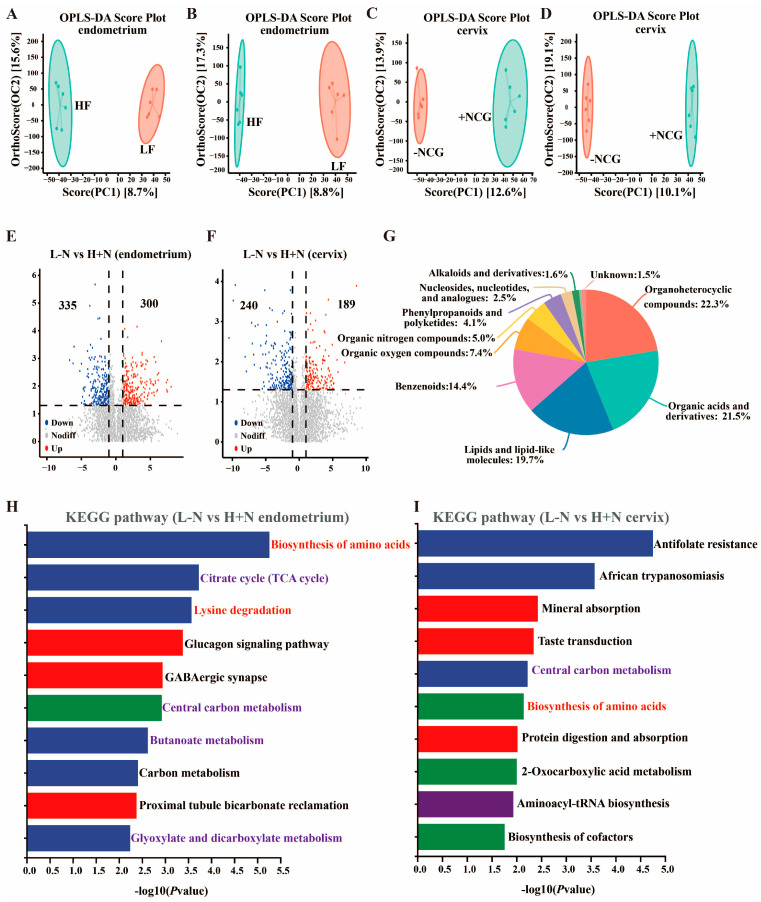
Effects of dietary fiber and N-carbamylglutamate on uterine metabolites. (**A**–**D**): OPLS-DA plots illustrated variations in metabolite composition between LF and HF groups, as well as between −NCG and +NCG groups in endometrium and cervix. (**E**,**F**): Volcano plots depicted differentially abundant metabolites between L − N and H + N groups in endometrium (**E**) and cervix (**F**). (**G**): Pie chart revealed compositional distribution of metabolites in the endometrium. (**H**,**I**): KEGG pathway enrichment analysis of differentially abundant metabolites between L − N and H + N groups in endometrium and cervix. OPLS-DA: Orthogonal Partial Least Squares—Discriminant Analysis; LF: low fiber; HF: high fiber; −NCG: without N-carbamylglutamate (NCG) supplementation; +NCG: with NCG supplementation; L − N: low-fiber diet without NCG; H + N: high-fiber diet supplemented with NCG.

The uterine microbiota serves as a key regulator of host metabolic networks through the production and transformation of bioactive metabolites. To assess effects of DF and NCG on metabolite profiles, untargeted metabolomics analysis was conducted on endometrium and cervix. Orthogonal partial least squares discriminant analysis revealed that HF and NCG exerted significant effects on metabolite composition in both endometrium and cervix (Figure 3A–D). Significant alterations in metabolite level were observed in endometrium and cervix following both DF and NCG treatments (Figure S2A–D). Notably, H + N resulted in substantial metabolic reprogramming, characterized by 335 downregulated and 300 upregulated metabolites in endometrium, and 240 downregulated and 189 upregulated metabolites in cervix compared to L − N group (Figure 3E,F). The identified uterine metabolites were primarily classified as organoheterocyclic compounds (22.3%), organic acids and derivatives (21.5%), lipids and lipid-like molecules (19.7%), benzenoids (14.4%), and organic oxygen compounds (7.4%).

Pathway enrichment analysis revealed that differentially abundant metabolites induced by DF were predominantly associated with carbohydrate metabolism-related pathways in endometrium, including central carbon metabolism in cancer, citrate cycle (TCA cycle), butanoate metabolism, and glyoxylate and dicarboxylate metabolism (Figure S2E), while lipid and nucleotide metabolism-related pathways were enriched in the cervix, including pyrimidine metabolism, arachidonic acid metabolism, and purine metabolism (Figure S2F). NCG-induced differentially abundant metabolites were primarily enriched in amino acid metabolism-related pathways in both endometrium and cervix (Figure S2G,H). Comparative analysis between L − N and H + N groups revealed that differentially abundant metabolites were significantly enriched in carbohydrate metabolism (including citrate cycle (TCA cycle), butanoate metabolism, glyoxylate and dicarboxylate metabolism, central carbon metabolism), and amino acid metabolism (including lysine degradation, biosynthesis of amino acids) (Figure 3H,I).

**Figure 4:**
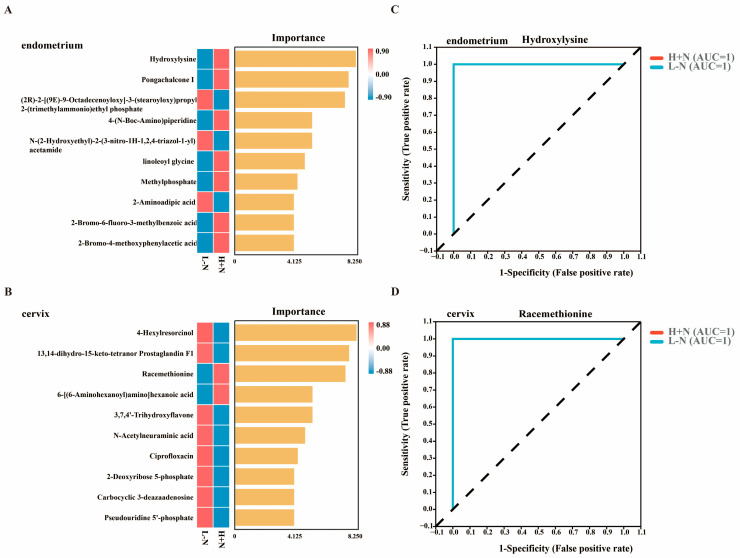
Biomarker identification and ROC analysis in endometrium and cervix following high fiber and N-carbamylglutamate supplementation. (**A**,**B**): Random forest models for biomarker identification in endometrium (**A**) and cervix (**B**). (**C**,**D**): Receiver Operating Characteristic (ROC) curve analysis of hydroxylysine (**C**) and racemethionine (**D**). AUC: area under the curve; L − N: low-fiber diet without N-carbamylglutamate; H + N: high-fiber diet supplemented with N-carbamylglutamate.

Random forest modeling and ROC curve analysis were employed to evaluate the discrimination ability of the key metabolites. Hydroxylysine and racemethionine were identified as characteristic metabolites representing the metabolic differences between L − N and H + N groups in endometrium and cervix, respectively (Figure 4A–D). Above findings demonstrated that DF and NCG supplementation, individually and in combination, induced comprehensive metabolic reprogramming in uterine tissues through modulation of carbohydrate, lipid, and amino acid metabolism pathways.

### 3.4. Correlation Analysis Among Metagenomics, Metabolomics, and Serum Indicators

**Figure 5:**
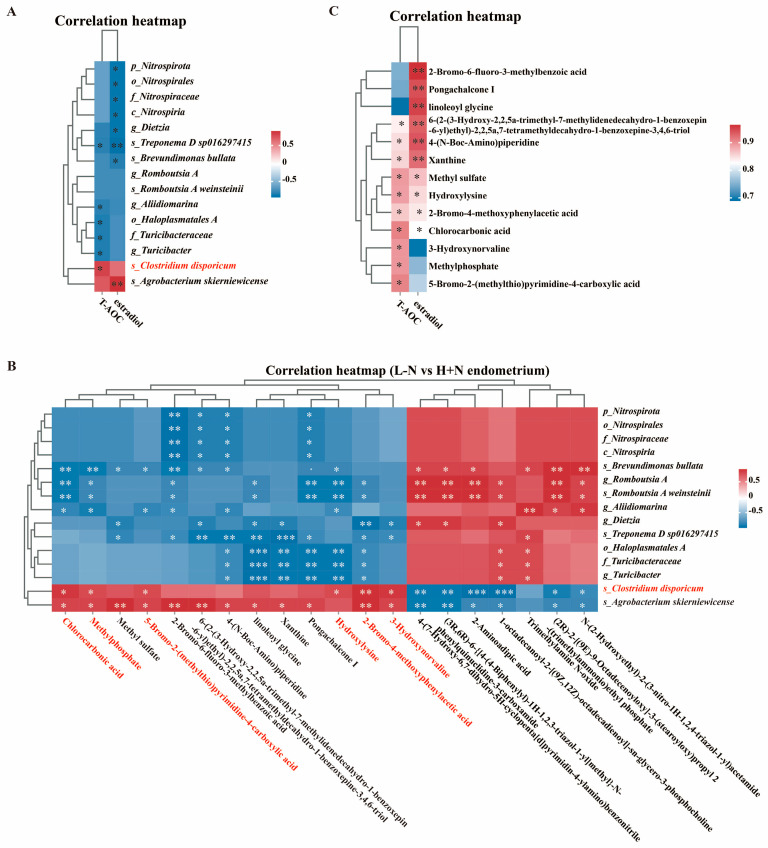
Correlation analysis among metagenomics, metabolomics data, and serum indicators. (**A**): Correlation analysis between top 15 differentially abundant microorganisms and serum indicators. (**B**): Correlation analysis between top 15 differentially abundant microorganisms and top 20 differentially abundant metabolites in endometrium. (**C**): Correlation analysis between differentially abundant metabolites and serum indicators. T-AOC: total antioxidant capacity; L − N: low-fiber diet without N-carbamylglutamate; H + N: high-fiber diet supplemented with N-carbamylglutamate. Asterisks indicate the level of statistical significance: * *p* < 0.05, ** *p* < 0.01, *** *p* < 0.001.

To delineate the associations between uterine microbial composition, metabolic profiles, and reproductive serum biomarkers, Spearman correlation analysis was employed to evaluate relationships among differentially abundant microorganisms, the differentially abundant metabolites, and differentially serum indicators. As shown in Figure 5A, differentially abundant microorganisms *Clostridium disporicum* and *Agrobacterium skierniewicense* exhibited significant positive correlations with T-AOC and estradiol levels, respectively. Subsequent investigations focused exclusively on *Clostridium disporicum*.

As shown in Figure 5B, *Clostridium disporicum* exhibited significant positive correlations with several organic acids and derivatives, including chlorocarbonic acid, methylphosphate, 3-Hydroxynorvaline, and hydroxylysine. Notably, the above metabolism displayed significant positive correlations with T-AOC and estradiol levels (Figure 5C). These findings underscored the interconnected roles of microbial composition and metabolite profiles in modulating serum indicators pertinent to reproduction.

### 3.5. Dietary Fiber and N-Carbamylglutamate Alter mRNA Expression Profile in Endometrium

**Figure 6:**
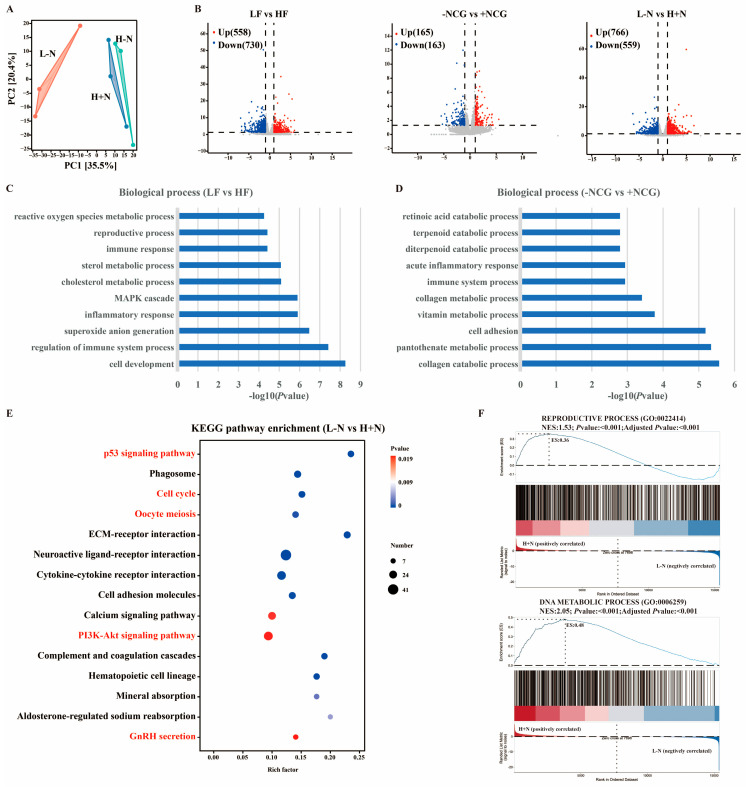
Effects of dietary fiber and N-carbamylglutamate on mRNA expression profiles in endometrium. (**A**): Principal component analysis (PCA) illustrated variations in mRNA expression among L − N, H − N, and H + N groups. (**B**): Volcano plot depicted differentially expressed mRNA between LF and HF groups, between −NCG and +NCG groups, as well as between L − N and H + N groups, respectively. (**C**,**D**): Biological enrichment analysis of differentially expressed mRNA between LF and HF groups (**C**), as well as between −NCG and +NCG groups (**D**). (**E**): KEGG pathway enrichment analysis of differentially expressed mRNA between L − N and H + N groups. (**F**): Gene Set Enrichment Analysis (GSEA) of differentially expressed mRNA between L − N and H + N groups. LF: low fiber; HF: high fiber; −NCG: without N-carbamylglutamate supplementation; +NCG: with N-carbamylglutamate supplementation; L − N: low-fiber diet without NCG; H − N: high-fiber diet without NCG; H + N: high-fiber diet supplemented with NCG; KEGG: Kyoto Encyclopedia of Genes and Genomes.

RNA sequencing analysis was performed to characterize endometrial gene expression patterns and identify molecular pathways through which DF and NCG improve reproductive efficiency. Principal component analysis demonstrated clear separation among the L − N, H − N, and H + N treatment groups (Figure 6A). Volcano plot analysis revealed significant alterations in gene expression levels following DF and NCG treatments (Figure 6B). Notably, H + N resulted in 559 downregulated and 766 upregulated mRNA in endometrium compared to the L − N group (Figure 6B).

Gene Ontology enrichment analysis revealed that differentially expressed genes induced by DF supplementation were significantly enriched in cell development, regulation of immune system processes, superoxide anion generation, mitogen-activated protein kinase cascade, and cholesterol metabolic processes (Figure 6C). In contrast, differentially expressed genes caused by NCG treatment were predominantly enriched in biological processes related to acute inflammatory response, immune system processes, collagen metabolic processes, vitamin metabolic processes, and cell adhesion (Figure 6D). KEGG pathway enrichment analysis indicated that differentially expressed genes between the L − N and H + N groups were predominantly associated with several critical signaling pathways, including p53 and PI3K-Akt signaling pathways, as well as processes involved in cell cycle regulation, oocyte meiosis, and gonadotropin-releasing hormone (GnRH) secretion (Figure 6E). Gene Set Enrichment Analysis revealed H + N regulated reproductive process and DNA metabolic process (Figure 6F). These results collectively demonstrated that DF and NCG supplementation induced transcriptional reprogramming in endometrial tissues.

### 3.6. Correlation Analysis Among Transcriptome, Metabolome, and Serum Indicators

**Figure 7:**
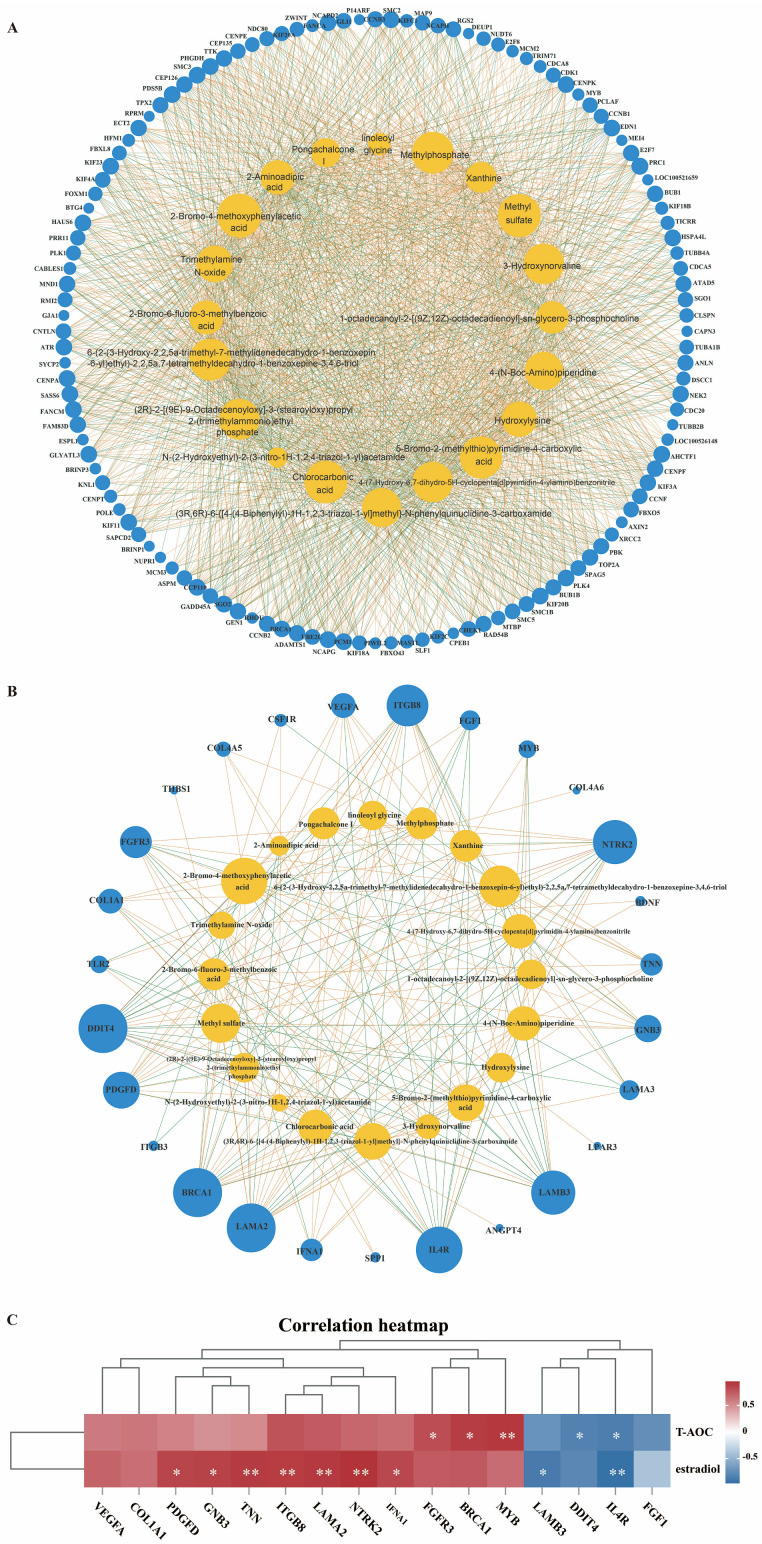
Correlation analysis among transcriptome, metabolomics data, and serum indicators. (**A**): Network diagram illustrated correlation between TOP20 differential metabolites and differential genes in cell cycle signaling pathway. (**B**): Network diagram illustrated correlation between TOP20 differential metabolites and differential genes in PI3K-Akt signaling pathway. (**C**): Correlation analysis between differential genes from PI3K-Akt pathways and serum indicators. Asterisks indicate the level of statistical significance: * *p* < 0.05, ** *p* < 0.01.

Pearson correlation analysis was performed on the top 20 differentially expressed metabolites and genes associated with the cell cycle and PI3K-Akt signaling pathway in the endometrium. Significant correlations were observed between selected metabolites and genes involved in the cell cycle (Figure 7A) as well as the PI3K-Akt signaling pathway (Figure 7B). The correlation coefficient and *p*-value were presented in the Supplementary Material (Excel). Specifically, chlorocarbonic acid showed significant positive correlations with *VEGFA*, *FGFR3*, *PDGFD*, *ITGB8*, *NTRK2*, *BRCA1*, *COL1A1*, and *LAMA2*, alongside significant negative correlations with *LAMB3*, *DDIT4*, *FGF1*, and *IL4R*. Methylphosphate exhibited significant positive correlations with *VEGFA*, *FGFR3*, *PDGFD*, *BRCA1*, *COL1A1*, *COL4A5*, and *LAMA2*, alongside significant negative correlations with *TLR2*, *DDIT4*, *FGF1*, and *IL4R*. Hydroxylysine exhibited significant positive correlations with *IFNA1*, *ITGB8*, *TNN*, *NTRK2*, *BRCA1*, *MYB*, and *LAMA2*, as well as significant negative correlations with *LAMB3*, *DDIT4*, and *IL4R*. 3-Hydroxynorvaline showed significant positive correlations with *VEGFA*, *FGFR3*, *BRCA1*, *COL1A1*, *COL4A5*, and *LAMA2*, alongside significant negative correlations with *DDIT4* and *FGF1*.

Further correlation analysis revealed that genes in the PI3K-Akt signaling pathway, such as *FGFR3, BRCA1, IFNA1, TNN, PDGFD, GNB3, ITGB8, NTRK2, LAMA2*, and *MYB*, exhibited significant positive correlations with T-AOC and estradiol levels (Figure 7C). These findings indicated that the PI3K-Akt signaling pathway contributed to the modulation of reproductive performance by DF and NCG.

## 4. Discussion

Antioxidants contribute to embryo development and offspring growth by mitigating oxidative damage [27]. T-AOC in serum serves as a comprehensive indicator for assessing the overall antioxidant status of the organism. In the present study, DF and NCG supplementation significantly increased T-AOC content (Figure 1D). This finding was consistent with previous studies: NCG has been shown to attenuate oxidative stress by enhancing T-AOC and glutathione content [28]. The above finding suggested that improved antioxidant capacity, as evidenced by increased T-AOC, may contribute to enhanced reproductive performance in sows, potentially through reduction in oxidative burdens on reproductive tissues [27].

There has been a growing focus on uncovering microbial communities in various reproductive organs and elucidating their intricate interactions with host physiological processes [29,30,31]. Research on the reproductive tract microbiome has undergone a fundamental transformation over the past decades. Historically, the uterus was regarded as a sterile organ, and any detected bacteria were automatically linked to disease [32]. However, accumulating evidence has now identified distinct microbial communities within the uterus, which were dynamic across reproductive phases and played a vital role in successful reproductive outcomes [29,30,33]. In the present study, the microbiota in both endometrium and cervix was composed predominantly of Actinobacteriota, Proteobacteria, and Firmicutes A (Figure 2A), which was aligned with previous reports that have documented these phyla as the dominant phyla in female reproductive tract microbiome [33]. Furthermore, functional enrichment analysis revealed that microbiota in endometrium and cervix was primarily associated with metabolic processes, including carbohydrate metabolism, amino acid metabolism, energy metabolism, lipid metabolism, and nucleotide metabolism (Figure 2D). This suggested that the reproductive tract microbiota may act as a crucial mediator in maintaining local homeostasis, potentially influencing reproductive outcomes through its role in nutrient processing and the production of bioactive metabolites.

In the present study, high-fiber diet supplemented with NCG significantly increased abundances of *Streptomyces aureoverticillatus, Herbaspirillum huttiense*, and *Clostridium disporicum* (Figure 2E,F). Among these microbes, *Clostridium disporicumis* warrants particular attention, as several species within the *Clostridium* genus have been shown to possess probiotic properties, including antioxidant and anti-inflammatory activities [34]. Furthermore, the chemoorganotrophic nature of *Clostridium* enables them to metabolize complex substrates, generating beneficial short-chain fatty acids such as acetate, propionate, and butyrate [26]. These microbial metabolites are modulators of host immune responses [26]. Importantly, prior research indicated that higher relative abundances of *Clostridium* species were associated with sows displaying superior reproductive performance [35], suggesting a potential link between uterine microbiota composition and enhanced reproductive outcomes. Complementing these microbial findings, network analysis in the present study revealed that changes in KO within the endometrium and cervix were primarily concentrated in key pathways including metabolism, cell cycle regulation, and mismatch repair (Figure 2G,H). The mismatch repair system is a critical defender against genomic instability and oxidative stress, often triggered by environmental stressors and DNA replication errors [36]. The observed differential KO may reflect variations in cellular stress levels or metabolic demands, which could subsequently affect uterine responses to reproductive challenges. Furthermore, these results supported existing studies linking greater reproductive capacity to higher metabolic burden, likely from increased oxidative stress and energy demands [37].

Microbiota modulates host homeostasis through a diverse array of metabolites [38]. The current study found that significantly increased metabolites in the uterus induced by H + N intervention were predominantly enriched in various amino acid metabolisms and energy metabolism (Figure 3H,I), which was consistent with previous studies [10,39]. Notably, ROC curve analysis revealed that hydroxylysine exhibited perfect discriminatory performance between the L − N and H + N groups (Figure 4C), indicating that H + N administration significantly altered the abundance of hydroxylysine. Hydroxylysine, an amino acid produced through lysine hydroxylation, serves as a critical component of collagen [40]. This modified amino acid plays a fundamental role in stabilizing collagen fiber cross-linking and facilitating triple helix structure formation, thereby maintaining the mechanical integrity and elasticity of connective tissues [41]. In the female reproductive system, collagen is widely distributed across tissues, including the endometrium, cervix, ovaries, and placenta [42]. A potential mechanism underlying the significant increase in hydroxylysine in the H + N group involves the action of short-chain fatty acids (SCFAs). SCFAs are produced via microbial fermentation of DF and may indirectly promote collagen metabolism [43]. Concurrently, NCG supplementation potentially provides more abundant substrates and optimizes the metabolic environment for collagen synthesis (containing hydroxylysine) by enhancing nitrogen metabolism and overall protein synthesis efficiency [44]. Of particular interest, *Clostridium disporicum* demonstrated significant positive correlations with hydroxylysine. Furthermore, both *Clostridium disporicum* and hydroxylysine exhibited significant positive correlations with T-AOC and estradiol levels (Figure 5A,C). These findings suggested that *Clostridium disporicum* may enhance systemic antioxidant status through modulation of hydroxylysine metabolism [34]. The improvement in redox balance potentially created a more favorable microenvironment for estradiol to exert their normal physiological function.

Transcriptomics analysis was performed to investigate the molecular mechanisms through which DF and NCG enhance reproductive efficiency. In the present study, the synergistic effects of the combined high-fiber diet and NCG intervention were predominantly mediated by phosphatidylinositol 3-kinase (PI3K)-Akt signaling pathway (Figure 6E), which is a key regulator involved in antioxidant [45], metabolism [46], immune [47], and reproduction [48]. In alignment with our findings, previous studies have demonstrated that DF supplementation exerted antidiabetic effects by activating the PI3K-Akt signaling cascade [49]. Similarly, NCG supplementation has been shown to enhance reproductive outcomes by promoting phosphorylation and activation of the PI3K pathway [50]. Notably, significant positive correlations were identified between hydroxylysine and the expression of genes associated with the PI3K-Akt signaling pathway (Figure 7B). These correlations suggested that hydroxylysine may function as an upstream modulator, potentially influencing PI3K-Akt activation through receptor-mediated signaling mechanisms or epigenetic regulation of gene expression. Activation of the PI3K-Akt pathway enhances antioxidant capacity [45], thereby maintaining uterine homeostasis by improving endometrial receptivity, promoting vascularization, and optimizing the local microenvironment for embryo implantation and pregnancy [50]. Together, these results suggested that combined supplementation with fiber and NCG improved uterine health and reproductive performance via the PI3K/Akt signaling pathway.

In summary, the objectives set for the present study were fully met. We aimed to investigate the synergistic effects of H + N on reproductive efficiency in gestating sows, and to reveal the underlying mechanisms via multi-omics analyses of uterine microbiota, metabolites, and transcriptome profiles. As demonstrated in the results, the combined H + N intervention significantly improved serum reproductive hormone levels and antioxidant capacity, reshaped the uterine microbial community (especially increasing the abundance of *Clostridium disporicum*), modulated key metabolites including hydroxylysine, and activated the PI3K-Akt signaling pathway in the endometrium. These changes collectively contributed to enhanced reproductive performance, which fully validated our research hypotheses and accomplished all stated objectives.

## 5. Conclusions

This study revealed that H + N is an effective nutritional strategy for enhancing reproductive performance in gestating sows. Multi-omics analyses revealed that H + N intervention significantly increased the abundance of *Clostridium disporicum* and metabolite hydroxylysine, concurrently activating PI3K-Akt signaling pathway. These changes were closely associated with serum estradiol and T-AOC levels in sows. The enhanced uterine antioxidant status helped alleviate oxidative stress, maintain endometrial homeostasis, and support normal folliculogenesis and hormone secretion, thereby creating a favorable uterine microenvironment for embryo implantation and pregnancy maintenance. Collectively, H + N modulated uterine microbiota–metabolite–transcriptome axis, strengthened antioxidant capacity, optimized reproductive hormone profiles, and ultimately improved the reproductive efficiency of sows. These findings provide a nutritional basis and mechanistic support for using high-fiber diets combined with NCG to improve reproductive outcomes in sow production.

## Figures and Tables

**Table 1:** Ingredients and chemical composition of basal diets (air-dry basis).

Items	Groups			
	H − N	H + N	L − N	L + N
Diet ingredients				
Corn	14.30	14.30	28.60	28.60
Hulled barley	45.00	45.00	45.00	45.00
Soybean meal	9.80	9.80	10.20	10.20
Rice bran meal	10.00	10.00	10.00	10.00
Soybean bran	9.00	9.00	0.00	0.00
Beet pulp	5.00	5.00	0.00	0.00
Soybean oil	0.90	0.90	0.00	0.00
Premix ^a^	3.00	3.00	3.00	3.00
Salt	0.40	0.40	0.40	0.40
Limestone	0.72	0.72	0.92	0.92
Dicalcium phosphate	1.65	1.65	1.65	1.65
Lysine hydrochloride	0.16	0.16	0.18	0.18
Methionine	0.01	0.01	0.00	0.00
Threonine	0.01	0.01	0.00	0.00
N-carbamylglutamate	0.00	0.05	0.00	0.05
Zeolite podwer	0.05	0.00	0.05	0.00
Total	100	100	100	100
Chemical composition ^b^				
DE, MCal·kg^−1^	3.06	3.06	3.06	3.06
CP, %	12.73	12.73	12.73	12.73
CF, %	7.46	7.46	3.73	3.73
NDF, %	20.09	20.09	13.71	13.71
ADF, %	9.85	9.85	5.07	5.07
Ca, %	0.76	0.70	0.65	0.73
AP, %	0.45	0.45	0.45	0.45
TD-Lys, %	0.60	0.60	0.60	0.60
TD-(Met + Cys), %	0.35	0.35	0.35	0.35
TD-Thr, %	0.42	0.42	0.42	0.42
TD-Trp, %	0.12	0.12	0.12	0.12

L − N: low-fiber diet without N-carbamylglutamate (NCG), L + N: low-fiber diet supplemented with NCG, H − N: high-fiber diet without NCG, H + N: high-fiber diet supplemented with NCG, CP: crude protein, CF: crude fiber, NDF: neutral detergent fiber, ADF: acid detergent fiber, Ca: calcium, AP: available phosphorus, TD-Lys: true digestible lysine, TD-(Met + Cys): true digestible methionine + cystine, TD-Thr: true digestible threonine, TD-Trp: true digestible tryptophan. ^a^ The premix provided the following per kg of diets: vitamin A 6000 IU, vitamin D3 3000 IU, vitamin E 30 IU, vitamin K3 2.5 mg, vitamin B1 3.0 mg, vitamin B2 4.0 mg, vitamin B6 4.0 mg, biotin 0.2 mg, folic acid 2.0 mg, nicotinic acid 20.0 mg, pantothenic acid 18.0 mg, Cu (as copper sulfate) 20.0 mg, Fe (as ferrous sulfate) 120 mg, Mn (as manganese sulfate) 50 mg, Zn (as zinc sulfate) 80 mg, KI (as potassium iodide) 0.5 mg, Se (as sodium selenite) 0.3 mg. ^b^ Contents of CP, CF, NDF, ADF, and Ca were determined experimentally, whereas the remaining nutritional components were calculated based on the Nutrient Requirements of Swine (GB/T 39235-2020 [25]).

## Data Availability

The metagenomics sequencing data are available in the NCBI Sequence Read Archive (SRA) under accession number PRJNA1378466. The metabolomics data are available in the MetaboLights database under accession numbers MTBLS13627 and MTBLS13635. The RNA-seq data are available in the NCBI Gene Expression Omnibus (GEO) under accession number GSE314489.

## Supplementary Materials

The following supporting information can be downloaded at: https://www.mdpi.com/article/10.3390/antiox15050542/s1.
