# Supplementary material for: High-Fiber Diet Supplemented with N-Carbamylglutamate Modulates Uterine Microbiota, Metabolites, and Transcriptome to Improve Reproductive Efficiency in Sows
Source: Antioxidants (Basel). 2026 Apr 24;15(5):542. doi: 10.3390/antiox15050542 (PMC13203796; doi:10.3390/antiox15050542)
Supplement: Supplementary file 1 [file antioxidants-15-00542-s001.zip › R1-E-supplementary material .pdf]

Figure S1. Dietary fiber and N-carbamylglutamate-induced modulations in uterine microbiota

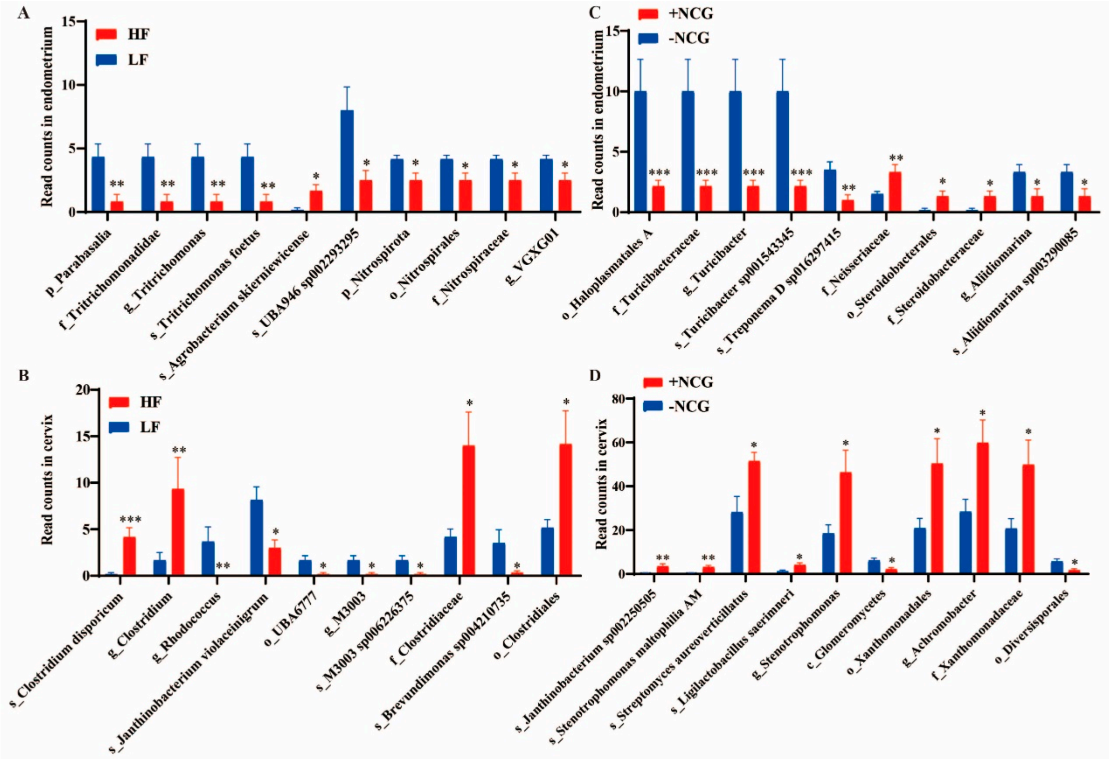

A-B: Read counts of differentially abundant microorganisms induced by dietary fiber in endometrium and cervix.

C-D: Read counts of differentially abundant microorganisms induced by N-carbamylglutamate in endometrium and cervix.

LF: low fiber; HF: high fiber; -NCG: without N-carbamylglutamate supplementation; +NCG: with N-carbamylglutamate supplementation.

Figure S2. Quantitative and functional analysis of differentially abundant metabolites induced by dietary fiber and N-carbamylglutamate

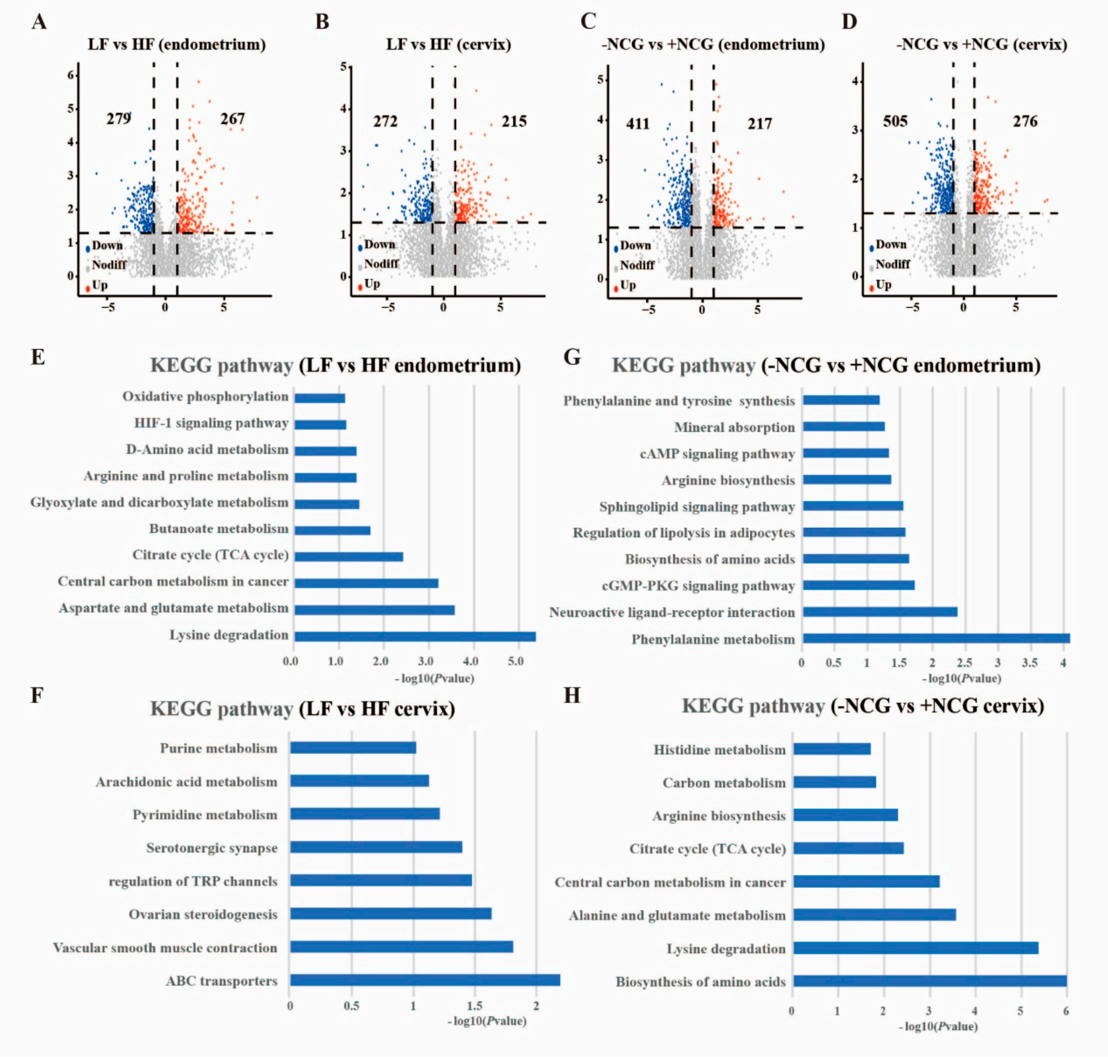

A-D: Volcano plots depicted differentially abundant metabolites between LF and HF groups, as well as between -NCG and +NCG group in endometrium and cervix.

E-F: KEGG pathway enrichment analysis of differentially abundant metabolites between LF and HF groups in endometrium (E) and cervix (F).

G-H: KEGG pathway enrichment analysis of differentially abundant metabolites between -NCG and +NCG groups in in endometrium (G) and cervix (H).

LF: low fiber; HF: high fiber; -NCG: without N-carbamylglutamate supplementation; +NCG: with N-carbamylglutamate supplementation.
